# The adiponectin promoter activator NP-1 induces high levels of circulating TNFα and weight loss in obese (*fa/fa*) Zucker rats

**DOI:** 10.1038/s41598-018-27871-7

**Published:** 2018-06-29

**Authors:** Juan Decara, Antonia Serrano, Francisco Javier Pavón, Patricia Rivera, Rocio Arco, Ana Gavito, Antonio Vargas, Juan A. Navarro, Ruben Tovar, Antonio J. Lopez-Gambero, Ana Martínez, Juan Suárez, Fernando Rodríguez de Fonseca, Elena Baixeras

**Affiliations:** 1 UGC Salud Mental, Instituto de Investigación Biomédica de Málaga (IBIMA), Universidad de Málaga-Hospital Universitario Regional de Málaga, Avda. Carlos Haya 82, Pabellón de Gobierno, 29010 Málaga, Spain; 2 Centro de Investigaciones Biológicas-CSIC, Ramiro de Maeztu 9, 28040 Madrid, Spain; 3 Departamento de Bioquímica y Biología Molecular, Facultad de Medicina, Universidad de Málaga, Málaga, Spain

## Abstract

Chronic NP-1 administration reduces body weight and hepatic steatosis despite induction of tolerance in adiponectin gene transcription with respect to the acute actions of this drug. This study explored the hypothesis that NP-1 could exert these effects through mechanisms independent of adiponectin. To this aim, we took advantage of the Zucker (*fa/fa*) rat model, which exhibits obesity, fatty liver and elevated leptin and adiponectin levels. Body weight and food intake were reduced after chronic NP-1 treatment. Plasma TNFα concentrations were elevated but no increase in adiponectin was found. Even so, NP-1 ameliorated fatty liver and corrected dyslipidemia by mechanisms probably associated with reduced feeding, transcription of *Cpt1* and down-regulation of *Hmgcr-CoA* expression. In brown fat tissue NP-1 increased *Dnmt1* (inhibitor of *Adipoq*) while it reduced *Ucp1* expression and heat production, which excludes thermogenesis as a mechanism of the NP-1 slimming effect. The anti-obesity action of chronic NP-1 administration might be mediated by TNFα, which is known to have anorectic actions in the hypothalamus and to regulate both *Dmnt1* and *Ucp1* expression in adipose tissues. This finding opens up the possibility of using NP-1-mediated TNFα-induced weight loss as an innovative treatment of complicated obesity under strict pharmacologic control.

## Introduction

**Figure 1:**
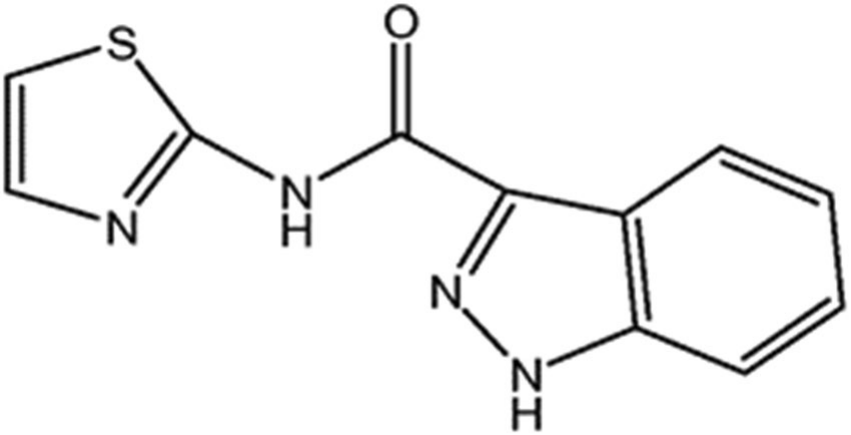
Basic structure of the N-(2-thiazolyl)-amide derivative NP-1 molecule (from patent WO 2009019202 A1).

The role of adiponectin in reducing food intake and decreasing body weight through its action on the hypothalamus has been established in rodents^1,2^. In addition, adiponectin is known to regulate glucose and insulin sensitivity as well as fatty acid oxidation in peripheral tissues through activation of the AMP-activated protein kinase (AMPK) and the peroxisome proliferator-activated receptor alpha (PPARα)^3,4^. Hypoadiponectinemia is normally associated with obesity and the metabolic syndrome, which includes insulin resistance, diabetes and non-alcoholic fatty liver disease (NAFLD)^4–7^. Failure in *adiponectin* transcription occurs through hypermethylation of its promoter^8^. Therefore, restoration of normal levels of adiponectin was held as a good clinical application with high potential in the treatment of obesity and diseases associated with the metabolic syndrome. In this regard, we recently reported a new thiazol-derived drug termed NP-1 (Fig. 1), described as an activator of the *adiponectin* promoter^9^. When administrated acutely, NP-1 demonstrated its ability to increase adiponectin levels and to ameliorate both hepatic steatosis and insulin resistance in a rat model of diet-induced obesity. Of note too was the fact that NP-1 also reduced both food intake and body weight in this diet-induced obesity model^10^. However, an apparent limitation of NP-1 after repeated administration was the induction of pharmacologic tolerance. However, despite this tolerance, the reduction of food intake and the slimming effects of NP-1 were maintained throughout the whole period of NP-1 administration^10^. These observations raised the question of whether NP-1 could exert its pharmacologic effects beyond just the activation of adiponectin signaling. To gain further insight into the mechanism of this novel class of drug, we took advantage of the model of obese Zucker rats that carry the spontaneous *fa* mutation that affects the extracellular domain of the leptin receptor, resulting in very low receptor sensitivity and therefore in leptin resistance. Leptin is an adipokine that contributes to regulating energy balance by inhibiting hunger^11–13^. Consequently, (*fa/fa*) Zucker rats exhibit hyperphagia and obesity induced by overfeeding, while displaying elevated levels of leptin^14,15^. Obese (*fa/fa*) Zucker rats also show evidence of marked liver steatosis, resulting in hypertrigliceridemia, hypercholesterolemia and hyperinsulinemia^16,17^. Interestingly, even though hypoadiponectinemia is a hallmark of obesity^5–7^, the obese (*fa/fa*) Zucker rats display high and sustained amounts of adiponectin in circulation^18^. Since obese Zucker rats show overfeeding in spite of the high amounts of adiponectin, it may be that adiponectin resistance has been established in the hypothalamus, thus further increasing the appetite in these animals. In fact, it has been reported that adiponectin and leptin act synergistically to potentiate body weight loss^1^, so functional failure of both hormones simultaneously could be expected to further potentiate over-feeding, and consequently obesity. Therefore, obese Zucker model provides us with a unique phenotype with which to test the effects of NP-1 beyond just adiponectin activation.

The aim of this study was to extend our previous investigations on the anorexic and slimming properties of NP-1 using the Zucker rat model. Here, we investigated whether chronic administration of NP-1 could exert its properties as a controller of food intake and lipid metabolism in a context of leptin and adiponectin resistance *in vivo*.

## Results

### Feeding behavior and body weight loss of lean and obese (*fa/fa*) Zucker rats after chronic NP-1 administration

**Figure 2:**
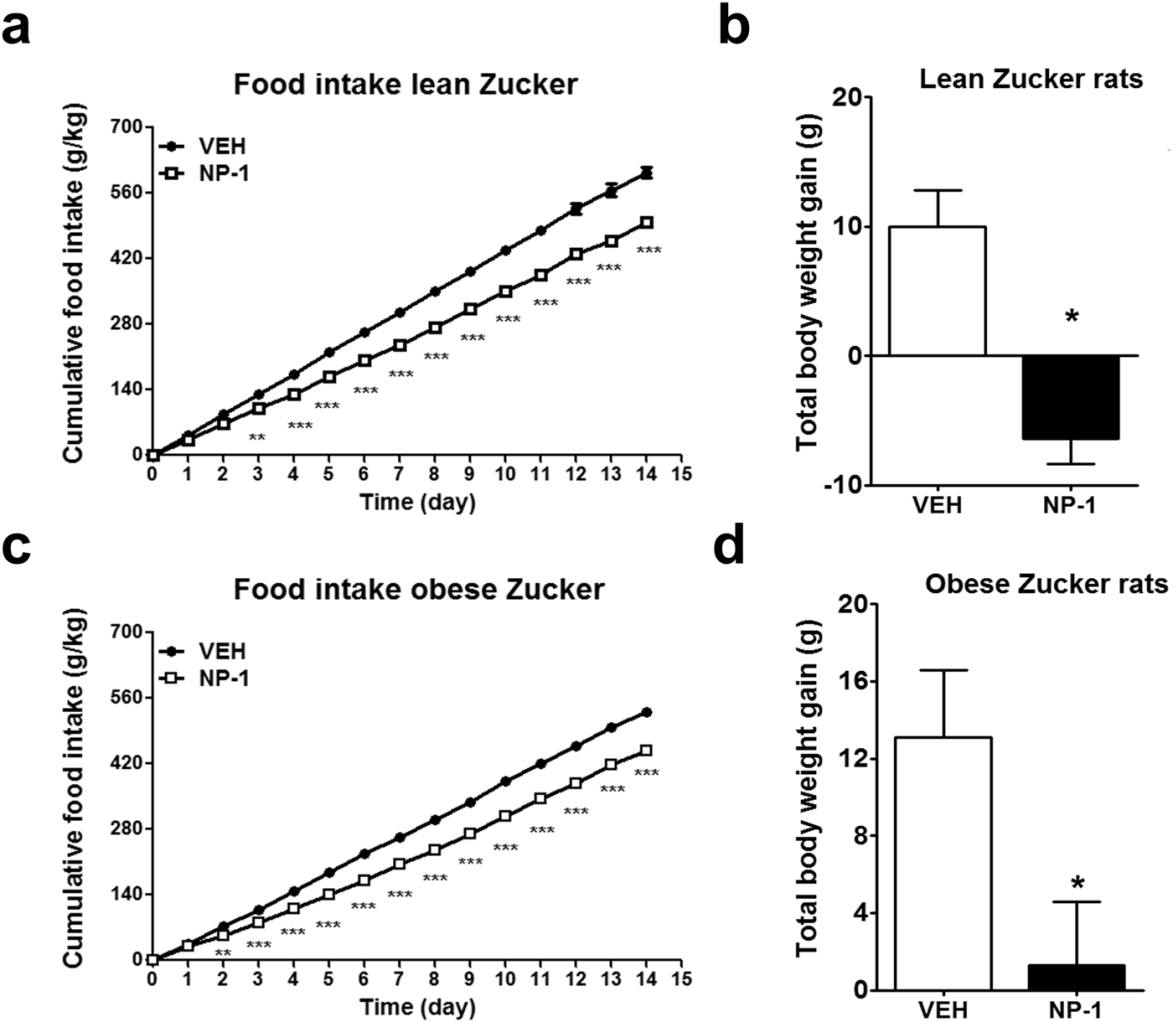
Effect of chronic NP-1 administration on cumulative food intake and total body weight gain in Zucker rats. After a 15-day exposure to vehicle (VEH) or NP-1 (5 mg / Kg, daily, i.p.) the cumulative food intake and total body weight gain were evaluated in the lean rats (**a**,**b**) and obese rats (**c**,**d**). Values are presented as the means ± SEM (n = 6–9 rats per group). Cumulative food intake data were analysed using two-way ANOVA (treatment and time) and a Bonferroni post hoc test for multiple comparisons (**a** and **c**). Differences in total body weight gain between VEH and NP-1 treated groups were analysed through unpaired, two tailed t-tests with Welch’s corrections (**b** and **d**). *P < 0.05, **P < 0.01 and ***P < 0.001 denote significant differences compared with the vehicle-treated group (VEH).

The effect of 15 days’ treatment with NP-1 or vehicle alone was examined daily regarding the cumulative food intake in the lean and obese (*fa/fa*) Zucker rats, fed standard laboratory chow. Compared with the respective vehicle-treated groups, chronic NP-1 treatment resulted in a significant reduction in food intake at all-time points with effect from as early as the third day of treatment in the lean rats (Fig. 2a) and the second day in the obese rats (Fig. 2b). The reduced food intake was accompanied by a significant loss in final body weight gain in lean and obese rats treated with NP-1 as compared with their vehicle-treated counterparts (Fig. 2c,d). Therefore, these observations indicate that chronic NP-1 administration reduced food intake and body weight in a context of leptin resistance, as is that of the obese Zucker rats^19,20^.

### Effect of chronic NP-1 administration on plasma levels of anorectic hormones

**Figure 3:**
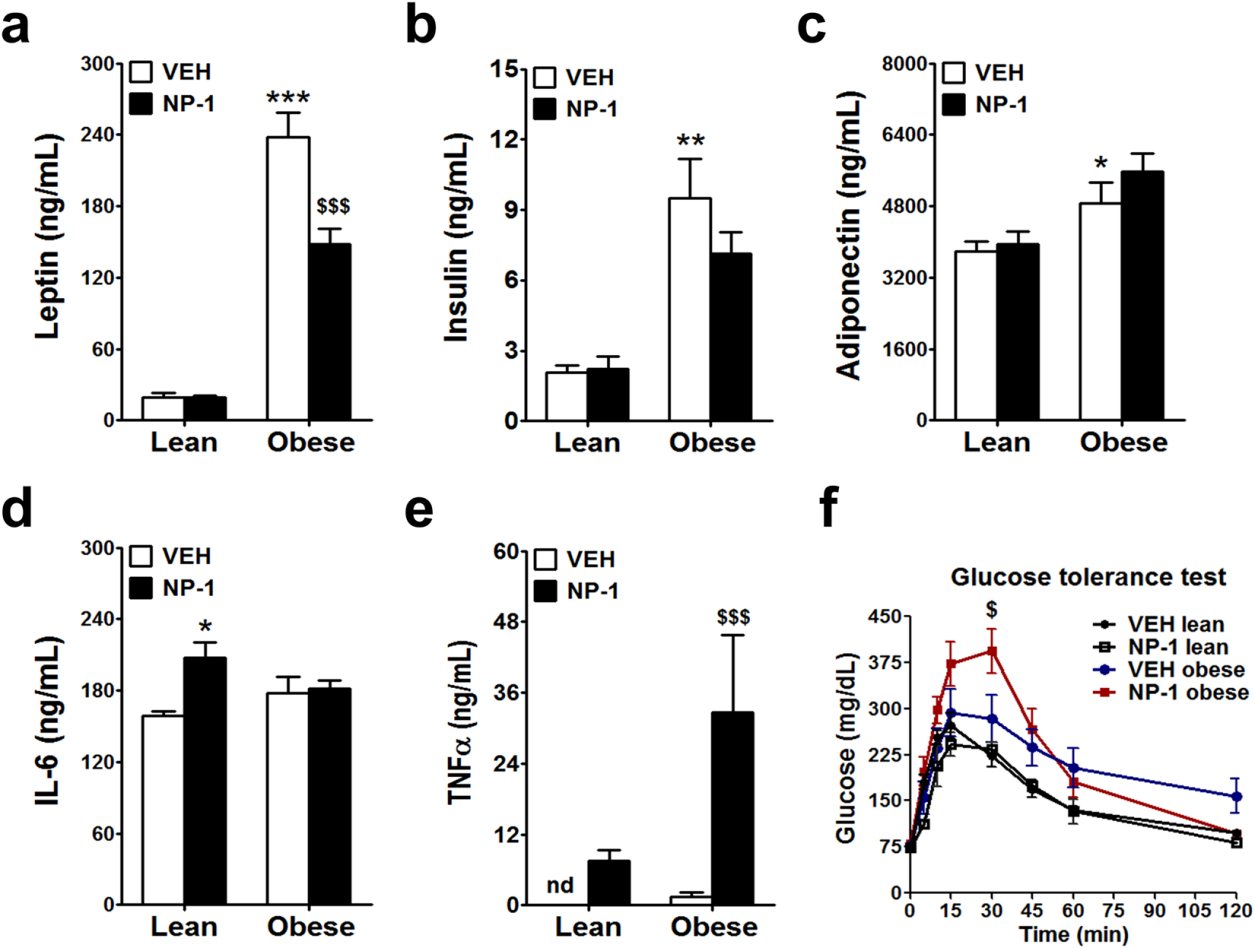
Effect of NP-1 on the plasma levels of cytokines that control food intake and glucose tolerance in Zucker rats. (**a**) leptin, (**b**) insulin, (**c**) adiponectin, (**d**) IL-6, (**e**) TNFα, were evaluated in the lean and obese rats after a 15-day exposure to vehicle (VEH) or NP-1 (5 mg/Kg, daily, i.p.). (**f**) Glucose tolerance test was performed at the end of the treatment. Animals were fasted for 18 h before they received an i.p. injection of glucose (2 g/ Kg body weight). Blood glucose concentrations were measured in blood drawn from the tail vein using a glucometer (AccuCheck, Roche, Germany) at times indicated in the figure. Values are presented as means ± SEM (n = 6–9 rats per group). Data were analysed using two-way ANOVA (treatment and genotype) and a Bonferroni post hoc test for multiple comparisons. *P < 0.05, **P < 0.01 and ***P < 0.001 vs VEH lean group. ^$^*P* < 0.05 and ^$$$^*P* < 0.001 vs VEH obese group.

We then evaluated the plasma concentration of the anorectic hormones leptin^11–13^ and adiponectin^1,2^, in the lean and obese rats after treatment with NP-1 or vehicle alone. As shown in Fig. 3a, the circulating levels of leptin in the vehicle-treated obese rats were higher than in the vehicle-treated lean rats, a phenomenon associated with decreased sensitivity of the leptin receptor in this strain and therefore with leptin resistance^11,19,20^. The baseline levels of leptin observed in the lean rats were not affected by NP-1 administration but in the obese group they were reduced markedly after treatment with NP-1 (Fig. 3a).

As insulin has also been considered a prandial satiety hormone^21^, we investigated the effects of chronic NP-1 treatment on the plasma levels of this hormone. The circulating insulin levels were significantly increased in the vehicle-treated obese rats compared with the matching lean group (Fig. 3b). This observation is compatible with the insulin resistance described in this obese strain^16,17^. Treatment with NP-1 did not affect significantly the insulin levels in either lean (Fig. 3b) or obese rats (Fig. 3b).

Adiponectin also decrease body weight and mediate anorexigenic effects^1,2^. In agreement with reported data^18^, the levels of this adipokine were significantly elevated in the plasma of obese rats treated with vehicle with respect to the levels found in their counterpart lean rats (Fig. 3c). Treatment with NP-1 did not change the plasma concentrations of adiponectin in either lean or obese rats compared to their vehicle-matched groups (Fig. 3c). This last result was, *a priori*, in contradiction with the supposed ability of NP-1 to up-regulate the *Adipoq* gene^9^, though it was in accordance with previous findings in which pharmacologic tolerance was suspected for NP-1 when administered for long periods of time^10^.

The proinflammatory cytokines IL-6 and TNFα can also act as cofactors that promote weight loss^22^, and given that these two cytokines are also expressed by adipose tissue^23^ we explored the impact of NP-1 treatment on plasma levels of these two cytokines in both the lean and obese rats. No important differences were observed regarding the circulating IL-6 levels between the vehicle-treated lean and obese rats (Fig. 3d). Chronic administration of NP-1 induced a significant increase of IL-6 levels in the lean rats whereas no effect was observed in the obese rats (Fig. 3d). However, very marked effects of NP-1 were observed regarding TNFα in plasma. Whereas the vehicle-treated lean rats showed undetectable levels of TNFα in plasma, the obese rats displayed detectable circulating levels of this cytokine (Fig. 3e). Chronic NP-1 treatment induced the expression of TNFα in plasma of the lean rats and increased notably the TNFα levels in the obese rats compared to the vehicle-treated group (Fig. 3e).

TNFα has been extensively reported to induce insulin resistance related to obesity^24^. In this concern, we also examined the effect of the chronic NP-1 treatment on blood glucose levels after glucose and insulin single administration in overnight fasted lean and obese rats. In the lean rats, the analysis of blood glucose levels over the entire time course of glucose tolerance test (GTT) showed no significant differences in between vehicle-treated and NP 1-treated rats, suggesting that chronic NP-1 administration had no significant impact in controlling the glucose levels in this strain (Fig. 3f). However, in the obese strain, repetitive NP-1 administration induced a significant delay in the clearance of glucose which resulted evident at 30 min of glucose post-injection as compared with the vehicle-treated obese group (Fig. 3f), as it might be expected from the high circulating TNFα levels. Nevertheless, this was a transient effect since the basal levels of glucose were recovered at 120 min post-injection paralleling the levels detected in the lean groups. In regard to the insulin tolerance test (ITT), the longer and lasting fall in blood glucose levels observed in obese rats vehicle-treated as compared with lean matched group (see Supplementary Fig. S1) reflects the insulin resistance in this obese Zucker strain. Treatment with NP-1 in obese rats attenuated the insulin intolerance, although this effect was transitory since glucose levels at 120 min become again equal to those found in the vehicle-treated obese group (see Supplementary Fig. S1).

In order to gain insight on the status of insulin resistance in NP-1-treated animals, we also examined the ability of chronic NP-1 administration to modulate insulin-mediated cell signaling in skeletal muscle. To this aim, we analysed the phoshorylation state of Akt (p-Akt). The western blot analysis showed no significant differences in the p-Akt/Akt ratio between vehicle-treated and NP-1-treated groups in both strain rats (see Supplementary Fig. S2). These last results indicate that the chronic NP-1 administration did not affect significantly the insulin mediating signaling in striate muscle.

### Effect of chronic NP-1 administration on the gene expression of *Adipoq* and *Dnmt1* in white and brown adipose tissues

**Figure 4:**
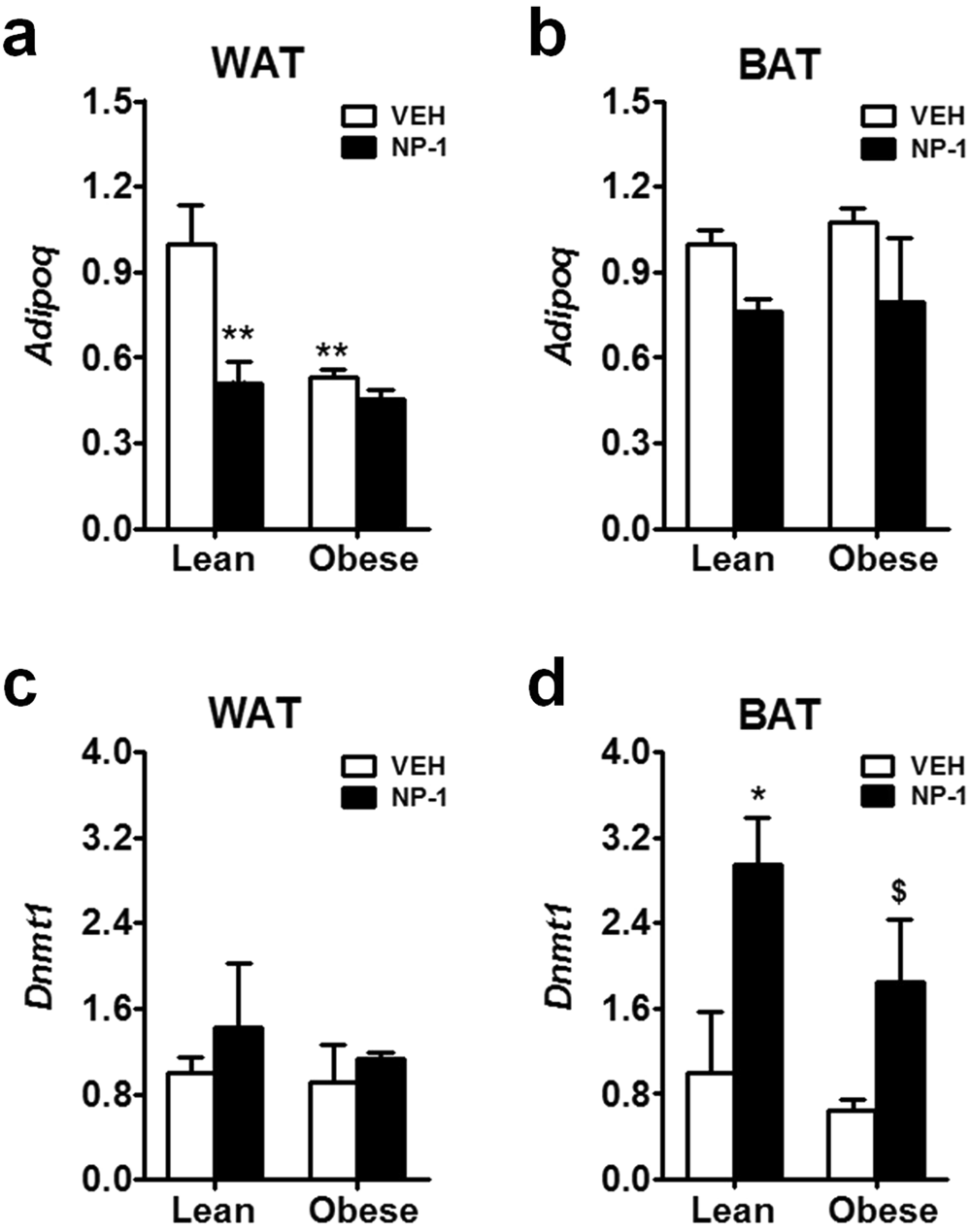
qPCR analysis of *Adipoq* and *Dnmt1* expression in adipose tissues of Zucker rats after NP-1 treatment. (**a**) Expression of *Adipoq* in WAT and BAT. (**b**) Expression of *Dnmt1* in WAT and BAT after chronic exposure to vehicle (VEH) or NP-1 (5 mg/Kg, daily, i.p.). Values are means ± SEM (6–8 animals per group). Two-way ANOVA and Bonferroni post-hoc test were used for data analysis: *P < 0.05; **P < 0.01 vs VEH lean group. ^$^*P* < 0.05 vs VEH obese group.

We analysed the expression of the *adiponectin* gene (*Adipoq*) after chronic treatment with NP-1 in the two main tissues that secrete adiponectin, WAT and BAT. After vehicle treatment, the *Adipoq* expression levels in the WAT of the obese rats were significantly lower than those found in the lean rats (Fig. 4a). This hypoadiponectinemia found in WAT of the obese rats was in agreement with their obesity status^5^. Intriguingly, chronic NP-1 treatment resulted in a significant decrease in *Adipoq* expression in the WAT of the lean rats but no change in the WAT of the obese rats, where the levels were already low from their hypoadiponectinemia (Fig. 4a). Regarding BAT, the expression of *Adipoq* in the vehicle-treated obese rats was not significantly different from that of the matching lean group (Fig. 4a). This observation infers that the secretion of adiponectin from BAT of the obese rats accounts for the maintained plasma levels of adiponectin in these rats (Fig. 3a). In agreement with the expected development of tolerance, treatment with NP-1 did not further increase *Adipoq* expression levels in the BAT of either the lean or the obese rats. In fact, a tendency to reduce this expression was observed with NP-1 treatment (Fig. 4a).

Failure in *Adipoq* transcription in obese subjects is in part attributed to hypermethylation of the *adiponectin* promoter mediated by DNA methyltransferase 1 (Dnmt1)^8^. Thus, in order to find an explanation for the last finding, we examined the expression levels of the *Dnmt1* gene in the adipose tissues studied under chronic NP-1 treatment. As shown in Fig. 4b, the expression levels of *Dnmt1* in WAT of NP-1 treated group did not differ significantly from the matched vehicle-treated groups. However, in the BAT from both the lean and the obese rats the chronic treatment with NP-1 significantly enhanced the expression of *Dnmt1* (Fig. 4b). Indeed, compared with their corresponding vehicle-treated groups, NP-1 up-regulated the *Dnmt-1* levels, 2.9-fold in the lean rats and 2.8-fold in the obese rats. Hence, this observation may explain why the levels of *Adipoq* and circulating adiponectin were not raised after chronic treatment with NP-1.

### Effect of chronic NP-1 administration on the AdipoR response in liver

**Figure 5:**
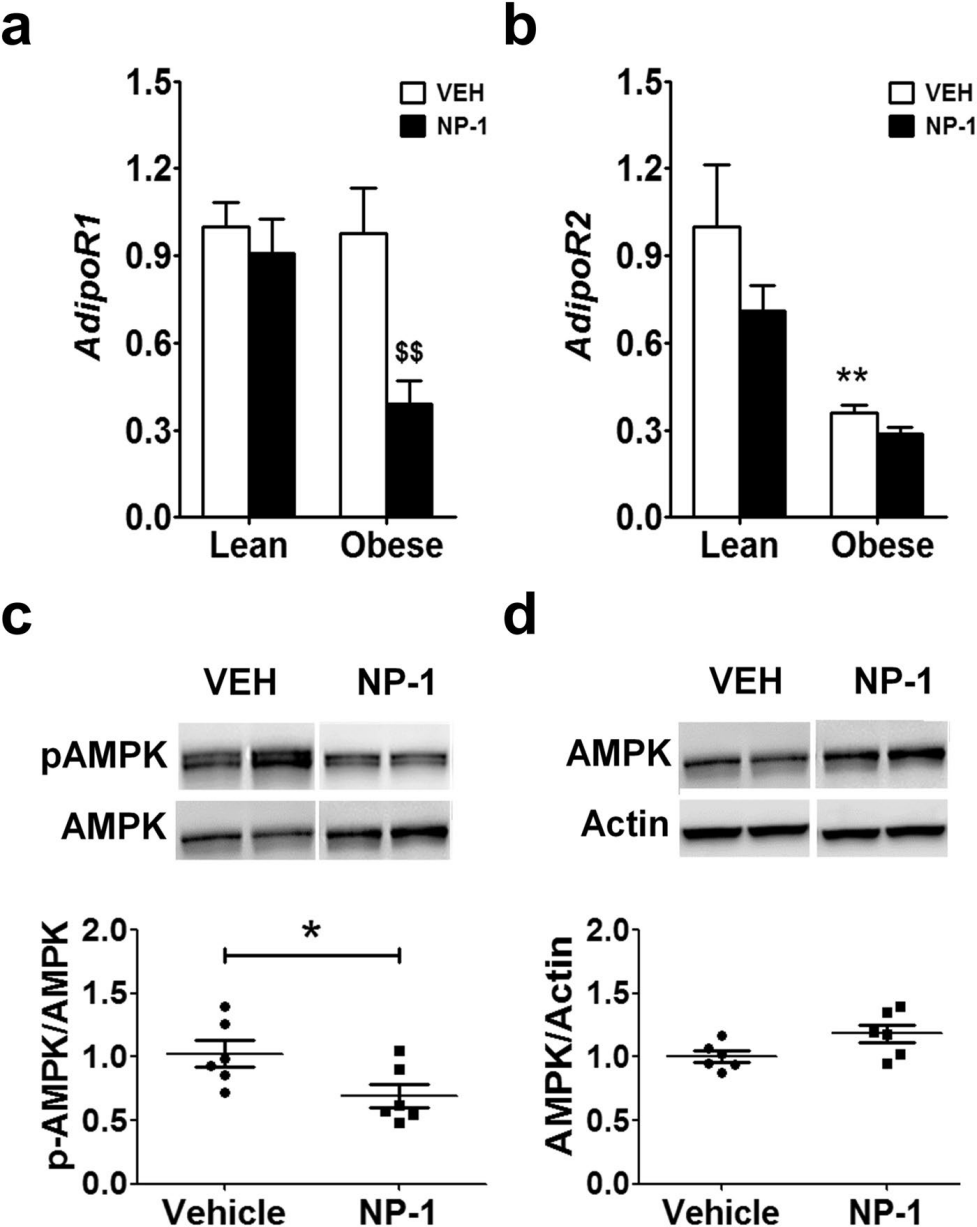
qPCR analysis of *AdipoR* isoforms expression and analysis of the phosphorylation state of the signaling mediator AMPK in liver of Zucker rats after NP-1 exposure. (**a**,**b**) Hepatic expression of *AdipoR1* and *AdipoR2* from lean and obese rats, treated chronically with vehicle (VEH) or NP-1 (5 mg/Kg, daily, i.p.). Values are means ± SEM (6–8 animals per treatment and genotype). Differences between groups were evaluated using two-way ANOVA and Bonferroni post-hoc test: **P < 0.01 vs VEH lean group. ^$$^*P* < 0.05 vs VEH obese group. (**c**,**d**) Representative western blot analysis (upper panels) and p-AMPK/AMPK ratio and AMPK/actin ratio (bottom panels) of liver samples from obese Zucker rats treated with vehicle (VEH) or NP-1. The blots show results from two out of six independent samples from each treatment group. The corresponding expression of actin is shown as a loading control per lane. All samples were derived at the same time and processed in parallel. The adjustment to digital images did not alter the information contained therein. Histograms below blots represent the corresponding densitometric values for either the p- AMPK/AMPK ratio or AMPK/actin ratio of the six independent samples from the obese rats. Differences between VEH and NP-1 treated groups were analysed through unpaired, two tailed t-tests with Welch’s corrections. *P < 0.05 vs VEH lean group.

Adiponectin activates the AdipoR1 and AdipoR2 isoforms, but in liver the isoform receptor AdipoR2 is mainly used by adiponectin^25^. The activation of AdipoR isoforms promotes glucose transport and inhibition of gluconeogenesis by activating AMP kinase (AMPK). In addition, AdipoR isoforms also mediate the PPARα pathway, thus favoring the β-oxidation of fatty acids and reduction of inflammation^25,26^. Since liver is a main target tissue for adiponectin actions^25^, we next explored the status of the hepatic AdipoR expression and its mediating signaling pathway after chronic NP-1 treatment. NP-1 treatment significantly down-regulated the hepatic expression of *AdipoR1* in the obese rats with respect to the levels found in their matching vehicle-treated group and the corresponding lean groups (Fig. 5a). The obese rats showed a significant down-regulated expression of *AdipoR2* after vehicle treatment, and administration of NP-1 did not return the levels to normal (Fig. 5b). The analysis of the phoshorylation state of the AMPK (p-AMPK) protein was focused in the liver samples from the obese strain. The analysis revealed that repetitive exposure to NP-1 produced a reduction in the p-AMPK/AMPK ratio (Fig. 5c). Examination of the AMPK/actin ratio from all samples showed no significant changes in the AMPK total content between the vehicle- and NP-1-treated groups (Fig. 5d). Together, these observations indicate that chronic NP-1 treatment promoted pharmacologic tolerance with a negative effect on both the expression of *AdipoR1* and AdipoR-mediated signaling in the liver of the obese rats.

### Fat content in liver of obese (*fa/fa*) Zucker rats is reduced after chronic NP-1 administration

**Figure 6:**
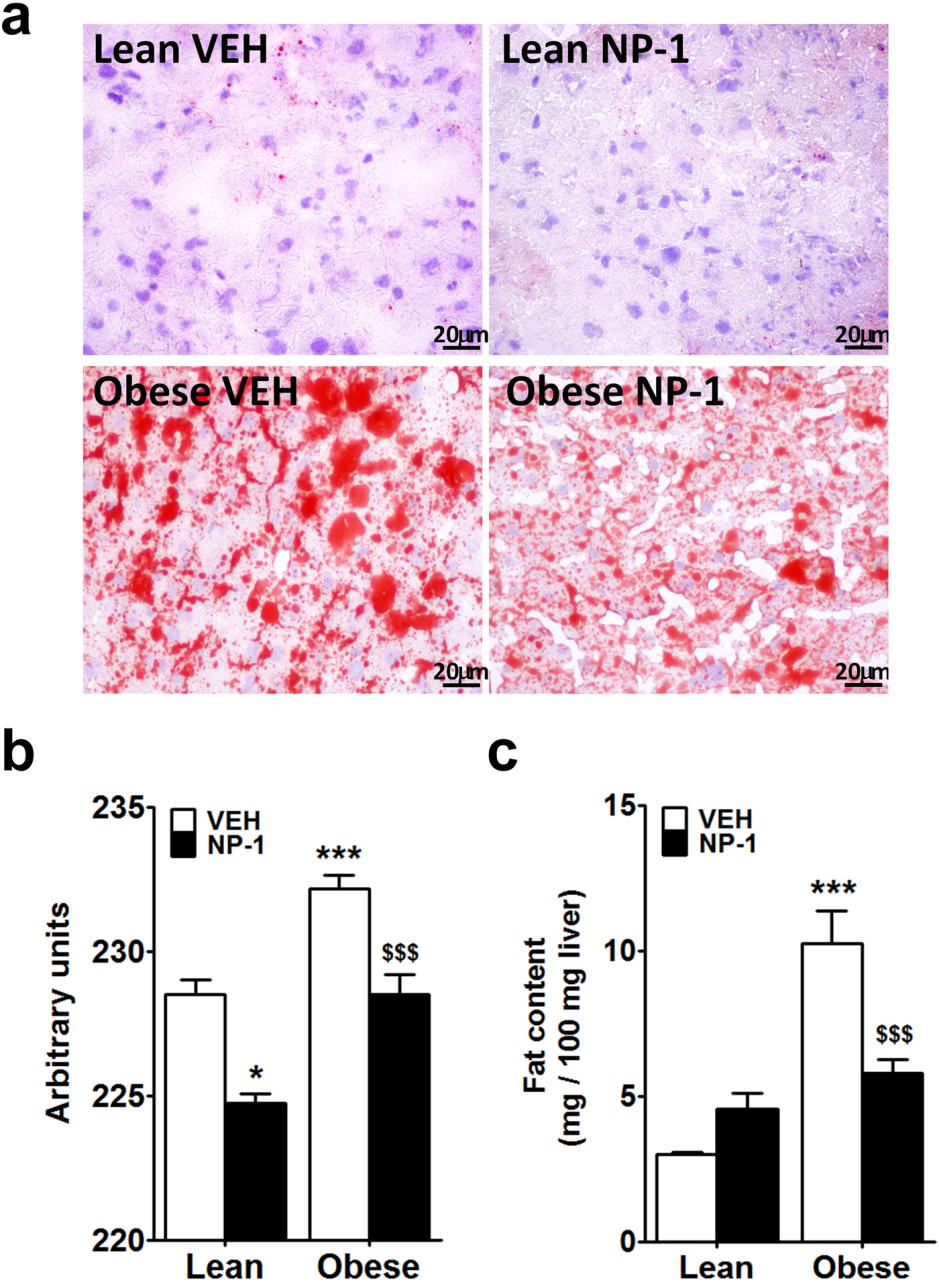
Effect of the chronic NP-1 treatment (5 mg/Kg, daily, ip) on the hepatic lipid content in lean and obese Zucker rats. (**a**) Representative images of liver fat content with Oil Red O staining. (**b**) Quantification of Oil Red O staining. (**c**) Measurement of the total fat content in the liver. Data are means ± SEM per group (n = 6–8). Differences between groups were evaluated using two-way ANOVA and Tukey post-hoc test: *P < 0.05 and ***P < 0.001 vs VEH lean group, ^$$$^*P* < 0.001 vs VEH obese group.

Based on the above results we reasoned that chronic NP-1 treatment should worsen the liver steatosis in the obese Zucker rats. Accordingly, the effects of the chronic NP-1 treatment on the hepatic lipid content were analysed. The liver sections were stained with oil red O (Fig. 6a). The staining showed some isolated patchy red staining in liver sections from the lean group whereas liver sections from the obese group displayed a marked increase in fat droplet content, compatible with severe steatosis (Fig. 6a). These observations were confirmed by quantification of the staining (Fig. 6b). Surprisingly, the chronic NP-1 treatment induced a significant reduction in the lipid droplet content in liver samples, especially from the obese rats (Fig. 6a,b).

The hepatic fat content was also assessed in all groups (Fig. 6c). Consistent with the oil red staining results, the liver from the vehicle-treated obese rats showed a significantly higher fat content than the matched lean group (Fig. 6b). After chronic NP-1 treatment the high hepatic fat content in the obese rats was significantly reduced while no significant effects were detected in the NP-1-treated lean rats (Fig. 6b).

### Chronic NP-1 administration ameliorates dyslipidemia in (*fa/fa*) Zucker rats

**Figure 7:**
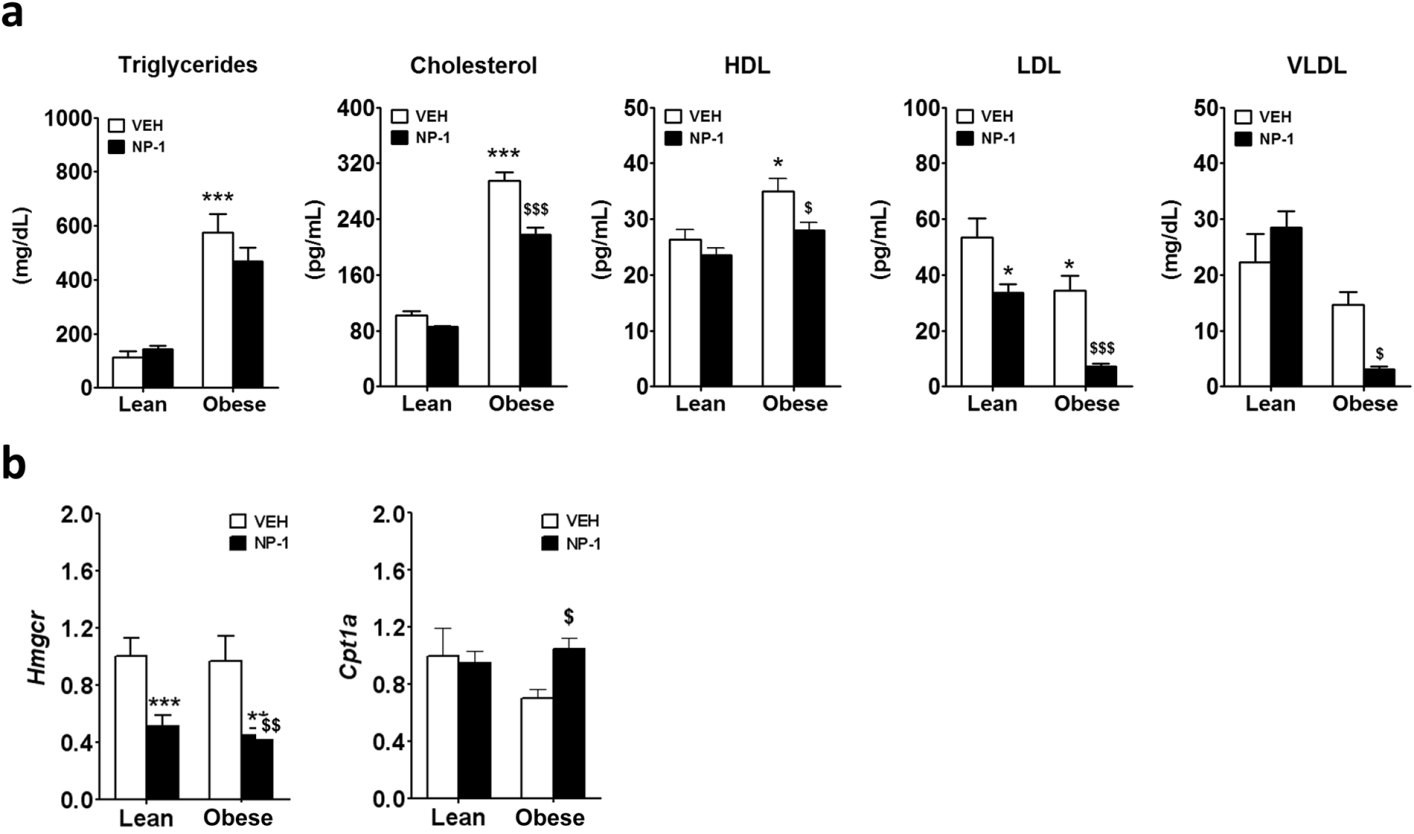
Effect of NP-1 on the plasma levels of metabolites and hepatic genes that control dyslipidemia in Zucker rats. (**a**) Effect of NP-1 on plasma lipid metabolites: triglycerides, cholesterol, HDL, LDL and VLDL measured in lean and obese Zucker rats after exposure to vehicle (VEH) or NP-1 (5 mg/Kg, daily, ip) for 15 days. (**b**) qPCR analysis of hepatic *Hmgcr* and *Cpt1a* expression in lean and obese Zucker rats after prolonged treatment with vehicle (VEH) or NP-1 (5 mg/Kg, daily, ip). The values are means ± SEM (6–8 animals per group). Differences between groups were evaluated using two-way ANOVA and Tukey post-hoc test: *P < 0.05 and ***P < 0.001 vs VEH lean group. ^$^*P* < 0.05 and *$$$P* < 0.001 vs VEH obese group.

The above results prompted us to analyse the impact of prolonged treatment with NP-1 on the lipid parameters in plasma from the NP-1-treated groups compared with the vehicle-treated groups. For the lipid profile we measured total cholesterol, triglycerides, HDL, LDL, and VLDL. As expected, there were significantly increased levels of serum cholesterol, triglycerides, and HDL in the vehicle-treated obese rats compared to the vehicle-treated lean rats (Fig. 7a). LDL levels were lower in the obese rats, while no significant differences were found in VLDL levels between the vehicle-treated lean and obese groups (Fig. 7a). With regard to the parameters observed in the vehicle-treated obese group, treatment with NP-1 induced a trend to reduced triglyceride levels but a significant reduction in cholesterol levels (Fig. 7a). NP-1 treatment did not further ameliorate the HDL levels in the obese rats but significantly reduced their levels of LDL and VLDL (Fig. 7a). These data clearly indicate that the chronic administration of NP-1 ameliorated the plasma lipid profile in the obese rats. Moreover, this also paralleled the reduction in liver fat content observed in the obese group (Fig. 6). Additional analysis in the hepatic tissue regarding the expression levels of a cohort of genes involved in lipid metabolism revealed that the gene expression of the enzyme 3-hydroxy-3-methyl-glutaryl-coenzyme A reductase *(Hmgcr-CoA)* was significantly down-regulated by NP-1 treatment in both strains (Fig. 7b). Since this enzyme catalyzes the conversion of HMG-CoA to mevalonic acid, the rate-limiting step in cholesterol synthesis, the inhibition of its expression by NP-1 treatment may explain the inability of NP-1 to increase the amounts of circulating HDL (Fig. 7a). Likewise, the lower levels of LDL and VLDL in plasma of the obese rats treated with NP-1 paralleled the increased expression of the *Carnitine palmitoyltransferase I (Cpt1*) gene in liver (Fig. 7b). *Cpt1* is a PPARα target gene^27^ encoding an essential enzyme in the β-oxidation pathway of the long-chain fatty acids. Therefore, it is likely that up-regulation of the *Cpt1* in liver promotes the β-oxidation of fatty acids, thus decreasing circulating levels of LDL and VLD.

Several plasma biomarkers of liver injury were also analysed in samples from the vehicle- or NP-1- treated lean and obese rats. While obesity results on significant increased plasma levels of creatinine and the liver transaminases GGT, AST and ALT when compared with the corresponding lean group (see Supplementary Table 1), the same analysis showed that chronic exposure to NP-1 did not significantly affect the levels of these metabolites in plasma from the obese rats. On the whole, these observations indicated that the NP-1 treatment did not aggravate liver damage.

### Chronic NP-1 administration inhibits the expression of *Ucp1* in BAT

**Figure 8:**
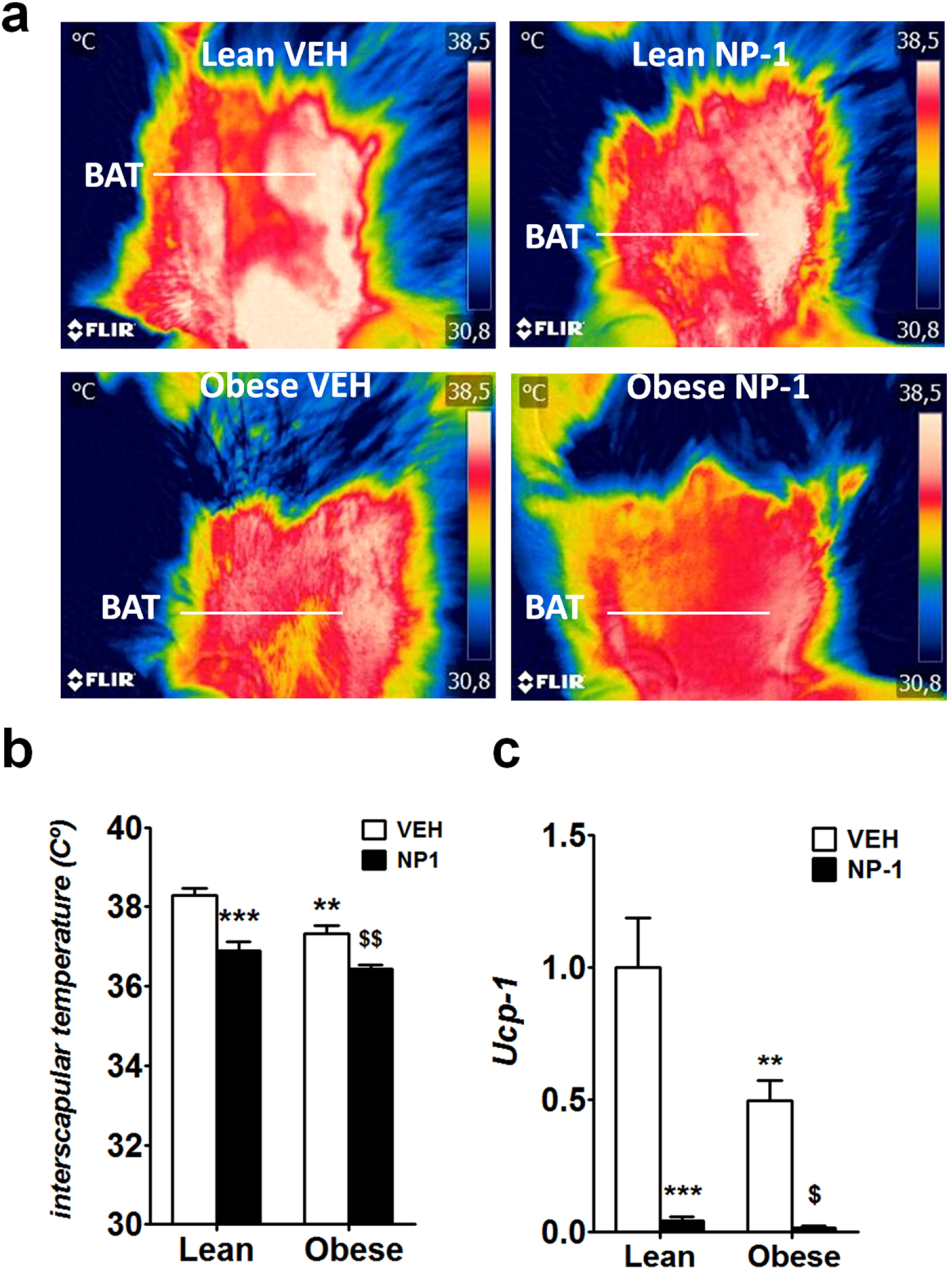
Effect of chronic exposure to NP-1 on the thermogenesis and on the expression of *Ucp1* in BAT of the Zucker rats. (**a**) Representative infrared thermal images with quantification of interscapular temperature. Interscapular temperature surrounding BAT was recorded with an infrared camera and evaluated in the lean and obese rats after a 15-day exposure to VEH or NP-1 (5 mg kg-1, daily, ip) as depicted in the figure. (**b**) Histogram representing the average interscapular temperature. (**c**) qPCR analysis of *Ucp1* gene expression in BAT. The bars are the means ± SEM (n = 8 rats per group). Data were analysed using two-way ANOVA (treatment and genotype) and a Tukey’s post hoc test for multiple comparisons. *P < 0.05, **P < 0.01 and ***P < 0.001 *vs* lean VEH group. ^$^*P* < 0.05 and ^$$^*P* < 0.01 vs obese VEH group.

The role of adiponectin in thermogenesis is controversial. Adiponectin was first reported to induce the up-regulation of uncoupling protein 1 (UCP1) in BAT, thus supporting its thermogenic potential and its mode of action to reduce body weight^1^. Another study has reported that adiponectin was found to inhibit *Ucp1* gene expression by suppressing β3-adrenergic receptor expression in BAT, therefore suppressing thermogenesis, an effect that was found to be independent of its two receptors, AdipoR1 and AdipoR2^28^. However, apart from these apparently opposing data, what is clear is that the obese status is related with reduced levels of *Ucp1*^29^; indeed, it has been shown that *Ucp1* ablation promotes obesity^30^. The results above described prompted us to analyse the impact of prolonged exposure to NP-1 on the thermogenesis and on the expression of *Ucp1* in BAT of the Zucker strains. The infrared thermal images of the skin temperature over interscapular BAT were quantified in both strains treated groups (Fig. 8a). Compared with the lean strain, the obese rats vehicle-treated showed a marked decrease in temperature surrounding interscapular BAT (Fig. 8a,b), which is compatible with the obese status^30^. The chronic administration of NP-1 in both lean and obese rats resulted in further reduction of the temperature in the same body area compared with that of the corresponding vehicle-treated rats (Fig. 8a,b). In agreement with reported data^29,30^, PCR analysis demonstrated that the expression of *Ucp1* was significantly decreased 2-fold in the obese group vehicle-treated as compared with the expression found in the matching lean group (Fig. 8c). The same analysis showed that chronic NP-1 exposure dramatically suppressed *Ucp1* expression in the BAT from both strains in regard to vehicle-treated matching groups (Fig. 8c). Therefore, on the whole these results indicate that the slimming effect of the chronic exposure to NP-1 cannot be explained by increased thermogenesis or augmented energy expenditure, suggesting that the improvement is derived mainly from reduced food intake.

## Discussion

Hypoadiponectimemia is a hallmark of obesity^5^, so we reasoned that chronic treatment with NP-1 could restore adiponectin levels and thereby re-establish satiety after eating. Nevertheless, both our previous studies as well as the present study showed that repetitive administration of NP-1 promotes pharmacologic tolerance to the enhanced circulating levels of adiponectin, so that NP-1 loses its ability to induce *adiponectin* expression. Nonetheless and surprisingly, the anorexia and weight loss as well as the beneficial effects on fatty liver prevailed throughout the chronic treatment^10^. These observations suggested that the effects of NP-1 could also be mediated by factors other than those mediated by the adiponectin itself.

In this study, we further analysed the *in vivo* effects of NP-1 on weight loss, anorectic actions and liver steatosis in the (*fa/fa*) Zucker model, a context of leptin resistance that promotes hyperphagia^11–13^. Chronic exposure to NP-1 reduced food intake and body weight in the obese Zucker rats and also decreased plasma levels of leptin in this strain. At present, the epigenetic modifications that control the *leptin* promoter in obesity remain unclear, but our findings are probably a reflection of a decrease in appetite and therefore a reduction in the need for high amounts of leptin. Compatible with pharmacologic tolerance regarding adiponectin bioactions, chronic administration of NP-1 reduced the expression of *Adipoq* in adipose tissues, particularly in WAT. We found also that chronic NP-1 treatment was paralleled by up-regulated expression of *Dnmt1*, especially in the BAT, which, in obese Zucker rats, is in fact the adipose tissue that supposedly sustains the adiponectin expression^18^. It is of note that NP-1 neither re-established the expression of *Adipoq* nor affected significantly the expression of *Dmnt1* in the WAT of the obese rats, thus indicating a specific action of NP-1 on BAT. Since NP-1 is an activator of the *Adipoq* promoter^9^, the *Dnmt1* up-regulation by NP-1 in BAT might explain, at least in part, the mechanism through which chronic exposure to NP-1 induces pharmacologic tolerance to the drug regarding adiponectin production in BAT.

We also found that treatment with NP-1 induced a negative regulation of the hepatic expression of *AdipoR1* in the obese rats that, together with the low expression of the *AdipoR2* isoform in this strain, could contribute strongly to reduced AMPK activity observed in the liver samples from NP-1-treated obese rats. Hence, these results further agree with a pharmacologic tolerance generated after prolonged exposure to NP-1 regarding adiponectin responses. However, in apparent contradiction with pharmacologic tolerance to the drug, we also found that despite the failure in AdipoR signaling, NP-1 treatment decreased the fat content in the liver of the obese rats, which is compatible with the observed amelioration of steatosis. Further, these relevant observations are in accordance with our previous results showing the beneficial effects of chronic exposure to NP-1 in reducing hepatic fat content in obese Wistar rats fed a high-fat diet^10^. The present findings therefore reinforce the notion that NP-1 could act through a mechanism other than adiponectin induction. In this regard, we show here that prolonged exposure to NP-1 is associated with an increase in the hepatic expression of *Cpt1* and lowered lipidemia in obese Zucker rats. This could provide an explanation for the decreased fat content in the liver since Cpt1 is one of the main enzymes involved in mitochondrial fatty acid oxidation^31^ whose activation might mediate the reduction of the hepatic fat content and circulating LDL levels observed after prolonged NP-1 exposure. Likewise, the reduced hepatic expression of the gene for the Hmgc-reductase enzyme, which is the bottleneck in cholesterol synthesis^32^, in rats treated for a long time with NP-1 could explain the inability of this drug to further increase HDL levels in plasma. In short, it is of note that the hyperlipidemia observed in the obese strain was counteracted by the treatment with NP-1. This finding, together with the fact that the values of transaminase as a whole did not increase after repeated administration of NP-1, make this treatment safety enough for liver.

A relevant finding in this study was the increased levels of circulating TNFα after chronic exposure to NP-1 in both strains, though particularly in the obese rats. High expression of TNFα has been related with insulin resistance in obesity^24^. Indeed, our results indicate that in the obese Zucker strain, which exhibits insulin resistance, the treatment with NP-1 aggravates transiently glucose intolerance, an effect that can be related to the high circulating TNFα. This additional insulin resistance seems to be not complete, since i) it is transiently overcome by exogenous administration of insulin (see Supplementary Fig. S1) and ii) it is not reflected in a significant reduction of Akt phosphorylation in the muscle (see supplementary Fig. S2) indicative of defective insulin signaling.

Interestingly, one of the genes under the control of TNFα is just that encoding for the Dnmt1 enzyme, which in turn is a specific inhibitor of *Adipoq* transcription^8^. Therefore, the increased levels of TNFα may explain the increased *Dnmt1* transcription in the BAT from animals treated with NP-1. The Dnmt1 would then lower the adiponectin tissue secretion rate even though this effect is not reflected in plasma levels. It might be that factors other than the adiponectin secretion rate from adipose tissue regulate the serum concentrations of adiponectin, such as adiponectin turnover and degradation^33^. Thus, for example, a study in humans has demonstrated that the half-life of human adiponectin is long (2.5 h) and its turnover rate is slow, so that its adipose tissue secretion rate would play a minor role in controlling circulating levels of adiponectin^33^. The same study showed that although the total body fat production rate is increased in obesity, adiponectin secretion from adipose tissue is reduced when expressed per tissue weight^33^. In addition, strongly correlated to its biological functions is the ability of full-length adiponectin to form homo-multimers in circulation, as also to be cleaved by proteolysis to produce the adiponectin globular head form^34,35^. Interestingly, the latter form appears involved in TNFα expression from macrophages and it has been suggested that the constant presence of high circulating levels of adiponectin renders macrophages tolerant to adiponectin and other pro-inflammatory stimuli^36^. In obese Zucker model, we do not rule out that after an overproduction of adiponectin induced by the first administrations of NP-1, an increase in TNFα could occur as a feedback mechanism to block the production of more adiponectin. Indeed, the results shown in this study concerning the expression of *Dnmt1* points to this possibility.

An additional and important point to be considered concerning TNFα is its close relationship with the phenomena of cachexia and its potent anorexigenic action in the hypothalamus^37–39^. Furthermore, TNFα promotes apoptosis of both preadipocytes and mature adipocytes, induces lipolysis and inhibits lipogenesis, and is an ideal player in the depletion of adipose tissue mass related with cachexia, which may be beneficial in extremely obese subjects^40^. Hence, we cannot rule out that TNFα overexpression could be one of the mechanisms through which NP-1 continues promoting the reduction of food intake and body weight. In addition, the reduced feeding potentially derived from a TNFα increase might account for the weight loss and improved metabolic profile in the obese rats.

In the (*fa/fa*) Zucker model, impaired thermogenesis is held to be the major cause of obesity, and the rats remain overweight when their food intake is restricted to that of their lean counterparts^29,41^. According to the latter, the present study shows that the interscapular temperature and BAT expression of *Ucp1* are lower in obese than in lean Zucker rats. However, even though the treatment with NP-1 reduced the body weight of the obese rats, both the interscapular temperature and the expression of *Ucp1*gene in BAT of lean and obese rat strains were reduced under chronic NP-1 treatment. These findings are compatible with the increased expression of TNFα after chronic NP-1 treatment since this cytokine is directly involved in the inhibition of *Ucp1* in BAT^42^. Ultimately, this indicates that the effect of NP-1 on weight loss cannot be attributed to increased thermogenesis or augmented energy expenditure, supporting the reduction in feeding as the major factor for decreasing liver steatosis and weight. Further research is needed to clarify whether the reduction in feeding is derived of the enhanced circulating levels of TNFα.

In summary, this study demonstrates that chronic exposure to NP-1 exerted anti-obesity and anorexic actions in a scenario with limited leptin and adiponectin bioactions. The results suggest that the chronic effects of NP-1 are also promoted by TNFα, which is known to have anorectic effects in the hypothalamus and permanently destroy fat cells. Thus, the repression of the *Adipoq* gene is probably mediated by the high levels of TNFα and its effects on Dnmt1 expression. Further studies are needed to determine the best pattern of NP-1 administration *in vivo* to improve the bioactions of adiponectin and regulate TNFα production in order to take advantage of its cachexic effect at the same time as avoiding its potentially harmful effects regarding its pro-inflammatory properties, muscle tissue loss and ability to induce insulin resistance^43^.

## Material and Methods

### Animals and ethics statement

Experimental procedures with animals were carried out in strict accordance with the recommendations in the European Communities directive 2010/63/EU and Spanish legislation (Real Decreto 53/2013, BOE 34/11370–11421, 2013) regulating the care and use of laboratory animals. The protocol was approved by the Ethics Committee for Animal Experiments of the University of Malaga. All studies involving animals are reported in accordance with the ARRIVE guidelines for reporting experiments involving animals^44^. All efforts were made to minimize animal suffering and to reduce the number of animals used. The experiments were performed on 8- to 9-week-old male obese (*fa/fa*) Zucker and male lean Zucker rats (Crl:ZUC-Leprfa; Charles River Laboratories, Barcelona, Spain). The animals were housed individually under a standard 12 h light-dark cycle in a room with temperature and humidity control. Water and rat chow pellets were provided *ad libitum* throughout the course of the present study.

### Drug preparation and dose administered

NP-1 was generously provided by Noscira (formerly Neuropharma, Tres Cantos, Spain). NP-1 was dissolved in a vehicle of 10% Tocrisolve-100 (Tocris Bioscience, Bristol, UK) and 90% physiologic saline and administered intraperitoneally (i.p.) at a dose of 5 mg/Kg, chosen based on its previously established anorectic properties^10^. Drugs were injected at a volume of 1 ml/kg.

### Feeding intake study in Zucker rats treated with NP-1

Animals (n = 6–8 in each group) received a daily i.p. injection of vehicle or NP-1 for 15 consecutive days. The amount of standard laboratory chow (3.02 kcal/g with 30 kcal% proteins, 55 kcal% carbohydrates, and 15 kcal% fat) eaten (kcal/kg body) and the body weight gain (g) were registered daily.

### Sample collection

The rats were sacrificed 2 h after the last dose. NP-1-treated and control animals were anesthetized with sodium pentobarbital (50 mg/Kg, i.p.), and blood and liver samples were collected. Blood was centrifuged (2100 g for 8 min, 4 °C) and the plasma kept for further analysis. Liver samples were divided into two pieces: one piece was flash-frozen in liquid N2, then stored at −80 °C until further analysis; and the other piece was fixed in 4% paraformaldehyde in 0.1 M phosphate-buffered saline (PBS) by immersion for 24 h and embedded in paraffin for further histologic and immunohistochemical analysis.

### Measurement of metabolites and hepatic enzymes in plasma

The following plasma metabolites were measured: glucose, triglycerides, total cholesterol, high-density lipoprotein (HDL), urea, uric acid, creatinine, and the hepatic enzymes alanine aminotransferase (ALT), aspartate aminotransferase (AST) and gamma-glutamyl transpeptidase (GGT). These metabolites were analysed using commercial kits according to the manufacturer’s instructions and a Hitachi 737 Automatic Analyser (Hitachi Ltd, Tokyo, Japan). Very low-density lipoprotein (VLDL) and low-density lipoprotein (LDL) were estimated with the Friedewald equations: VLDL = TG/5; LDL = TChol − [(TG/5) + HDL^45^.

The plasma levels of cytokines were determined with an enzyme-linked immunosorbent assay (ELISA) method using commercial kits: leptin and adiponectin ELISA kits (Abcam, Cambridge, UK); insulin ELISA kit (Mercodia, Uppsala, Sweden), IL-6 and TNF-α ELISA kit (Novex, by Life technologies, USA). All serum samples were assayed in duplicate within one assay, and results were expressed in terms of the particular standard hormone.

### Glucose and Insulin tolerance tests

Briefly, after chronic NP-1 treatment rats were food deprived for 18 h and given an i.p. injection of 2 g D-glucose /kg body weight for glucose tolerance test (GTT) or 0,75 UI insulin /Kg (Actrapid, Novonordisk Pharma) for insulin tolerance test (ITT). Blood samples were collected from the tail vein at 0, 5, 10, 15, 30, 45, 60 and 120 min after injection and glucose concentrations were measured with a commercially available glucometer (AccuCheck, Roche, Germany).

### Total fat extraction in liver

Extraction of total fat was performed as described^10^. Briefly, total lipids were extracted from frozen liver samples with chloroform-methanol (2:1, v/v) and butylated hydroxytoluene (0.025%, w/v) according to the Bligh and Dyer method^46^. The phase containing lipids was extracted after two centrifugation steps (2800 g, 4 °C for 10 minutes). Samples were dried by nitrogen and the liver fat content was expressed as a percentage of the tissue weight^10^.

### Oil red O staining

Liver samples were analysed for lipid and fat depots by oil red O staining. Frozen samples were cut into 30 µm-thick sections using a sliding microtome (Leica SM200R, Wetzlar, Germany) and fixed with 10% formal calcium. Sections were washed with distilled water and rinsed with 60% isopropanol. Then, sections were stained with freshly prepared oil red O (Sigma, St Louis, MO) working solution for 20 minutes (Oil red O stock stain: 0.5% of oil red O in isopropanol; Oil red working solution: 30 ml of the stock stain and 20 ml of distilled water). Sections were rinsed with 60% isopropanol, counterstained with Mayer’s hematoxylin, rinsed with tap water and mounted in aqueous media.

### RNA isolation and cDNA synthesis

Total RNA was extracted from tissue portions of liver, epididymal white adipose tissue (WAT) and dorsal interscapular brown adipose tissue (BAT) (100–300 mg) using the Trizol® method according to the manufacturer’s instructions (Gibco BRL Life Technologies). To ensure the purity of the mRNA sequences, RNA samples were isolated with an RNeasy Minelute Cleanup Kit (Qiagen), which included digestion with DNase I column (RNase-free DNase set, Qiagen), according to the manufacturers’ instructions. Total RNA was quantified using a spectrophotometer (Nanodrop 1000 Spectrophotometer, Thermo Scientific) to ensure A260/280 ratios of 1.8 to 2.0. Reverse transcription was carried out from 1 μg of RNA using the Transcriptor Reverse Transcriptase kit and random hexamer primers (Transcriptor RT, Roche Diagnostic GmbH). Negative controls included reverse transcription reactions that omitted the reverse transcriptase.

### Real-time Quantitative Polymerase Chain Reaction (qPCR) and Gene Expression Analysis

Real-time qPCR was performed following the criteria of the MIQE guidelines^47^. Polymerase chain reactions were carried out on the CFX96 Touch™ Real-Time PCR detection system (Bio-Rad, Hercules, CA) for each cDNA template, and amplified in 20 µl reaction volume containing 9 μl of cDNA (diluted 1/100) and 11 μl of master mix containing the primer (TaqMan, Life Technologies). The list of primers for the target rat genes *adiponectin* (*Adipoq*), *adiponectin receptor* (*AdipoR*), *DNA methyltransferase* 1 (*Dnmt1*), *Uncoupling protein*1 (*Ucp1*) can be found as Supplementary Table S2. All primers were obtained based on TaqMan® Gene Expression Assays and the FAM™ dye label format (Life Technologies). Each reaction was run in duplicate. Cycling parameters were 50 °C for 2 min to deactivate single- and double-stranded DNA containing dUTPs, 95 °C for 10 min to activate Taq DNA polymerase followed by 40 cycles at 95 °C for 15 sec for cDNA melting, and 60 °C for 1 minute to allow for annealing and the extension of the primers, during which fluorescence was acquired. Raw fluorescence data were submitted to the online Miner tool (http://www.miner.ewindup.info/) for calculation of Cq and efficiency values for each experimental set^48^. Cq values were converted into relative expression values taking into account amplification efficiencies, inter-run variations, and normalization factors by means of Biogazelle’s qbase^PLUS^ software (Biogazelle, Zwijnaarde, Belgium) using at least two reference rat genes: beta actin (*Actb*) and glyceraldehyde-3-phosphate dehydrogenase (*Gapdh*) for liver samples and ribosomal protein L9 (*Rpl19*) and Sp1 transcription factor (*Sp1*) for WAT and BAT samples (see Suplemmentary Table S2 on line). For all reference and target gene studies, two independent biologic samples of each experimental condition were evaluated in technical duplicates. Repeatability between replicates was accepted when the ΔCq value was ≤0.7. Finally, Calibrated Normalized Relative Quantity (CNRQ) values were exported from the qbase^PLUS^ software and investigated statistically.

### Protein extraction and Western blot analysis

Proteins from 50 mg of liver samples were extracted, separated in gradient SDS-PAGE gels and electroblotted onto nitrocellulose membranes and western blot analysis was performed as described^49^. Specific blotted proteins were detected with the corresponding primary rabbit antibody: anti-AMPK, anti-phospho-AMPK, anti-Akt, -anti-phospho-Akt (Cell Signaling Technology Inc. MA.; cat. number: #2532, #2535, #9272 and #9271, respectively); mouse anti-βactin (Sigma-Aldrich; cat. number: # A5316) and anti-Adaptin γ (Abcam, Cambridge, UK.;, cat. number #ab167153) followed by 1 h incubation with the corresponding secondary antibody: HRP-conjugated anti-rabbit IgG (H+L) or HRP-conjugated anti-mouse IgG (H+L) antibodies (Promega; cat. number W4011 and W4021, respectively). Specific protein bands were revealed using the enhanced chemiluminiscence detection system (Santa Cruz, Biotechnology Inc. CA, USA), in accordance with the manufacturer’s instructions. Images were visualized in an Autochemi-UVP Bioimaging System. Finally, bands were quantified by densitometry performed by ImageJ software (http://imagej.nih.gov/ij). Levels of specific proteins were normalized to actin levels for liver samples or adaptin levels for skeletal muscle samples.

### Thermal imaging acquisition and analysis

Interscapular temperature surrounding BAT was recorded with an infrared camera (Compact-Infrared-Thermal-Imaging Camera E60bx, FLIR, West Malling, Kent, UK) and analysed with a specific software package (FLIR Tools Software). For each animal/group (n = 8), three or four pictures were taken and analysed. The temperature surrounding BAT for one particular animal was calculated as the average temperature in a defined interscapular area (2 cm Ø) recorded by analysing those pictures^50^.

### Statistical analysis

Graph-Pad Prism 5.04 software was used to analyse the data. Values are represented as mean ± standard error of the mean (SEM) of 6–8 determinations for each experimental group according to the assay. The significance of differences within and between groups was evaluated by two-way analysis of variance (ANOVA) followed by post-hoc test for multiple comparisons. Alternatively, for comparisons between two groups, a Student *t*-test was also used. A *P*-value ≤ 0.05 was considered statistically significant.

### Data availability

All data generated or analyzed during this study are available from the corresponding author on reasonable request.

## Electronic supplementary material

Supplementary information
