# Supplementary material for: The adiponectin promoter activator NP-1 induces high levels of circulating TNFα and weight loss in obese (fa/fa) Zucker rats
Source: Sci Rep. 2018 Jun 29;8:9858. doi: 10.1038/s41598-018-27871-7 (PMC6026175; doi:10.1038/s41598-018-27871-7)
Supplement: Supplementary file 1 — Supplementary information [file 41598_2018_27871_MOESM1_ESM.pdf]

Supplementary Information

The adiponectin promoter activator NP-1 induces high levels of circulating TNFα and weight loss in obese (*fa/fa*) Zucker rats

Juan Decara, Antonia Serrano, Francisco Javier Pavón, Patricia Rivera, Rocio Arco, Ana Gavito, Antonio Vargas, Juan A. Navarro, Ruben Tovar, Antonio J. Lopez-Gambero, Ana Martínez, Juan Suárez, Fernando Rodríguez de Fonseca, Elena Baixeras.

<CONTENTS>

Supplementary Figures

Supplementary Tables

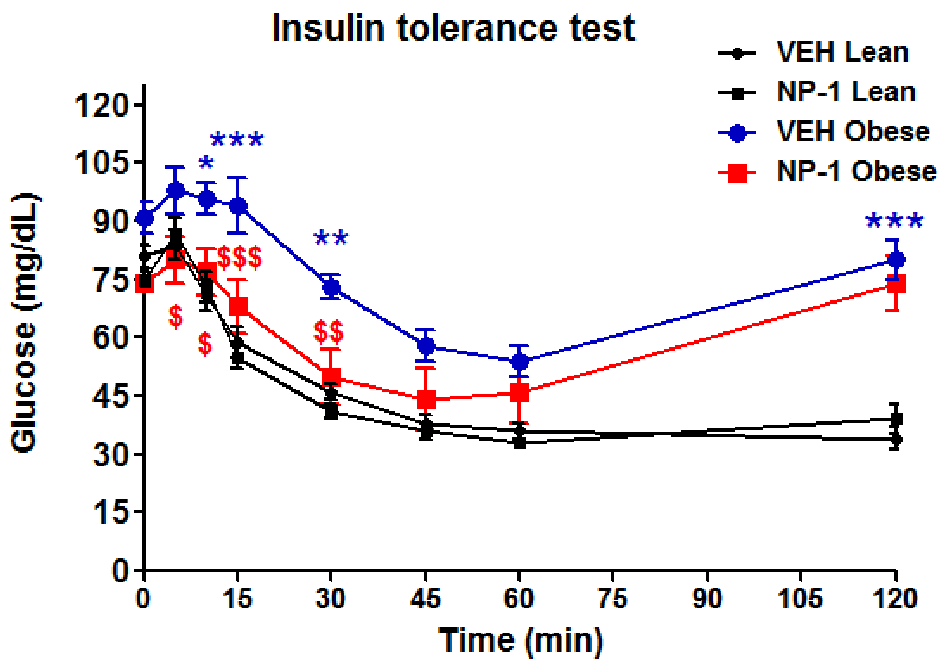

Supplementary Figure 1. Effect of chronic NP-1 treatment on insulin tolerance in Zucker rats. Insulin tolerance test was performed at the end of the treatment. Animals were fasted for 18 h before they received an i.p. injection of insulin (0.75 IU/Kg body weight). Blood glucose concentrations were measured in blood drawn from the tail vein using a glucometer at the times indicated in the figure. Values are presented as the means ± SEM (8 animals per treatment and genotype). Data were analysed using two-way ANOVA (treatment and genotype) and a Bonferroni post-hoc test for multiple comparisons. $^{*}P<0.05$, $^{**}P<0.01$ and $^{***}P<0.001$ VEH obese group *vs* VEH lean group. $^{\$}P<0.05$, $^{\$\$}P<0.01$ and $^{\$\$\$}P<0.001$ *vs* VEH obese group *vs* NP-1 obese group.

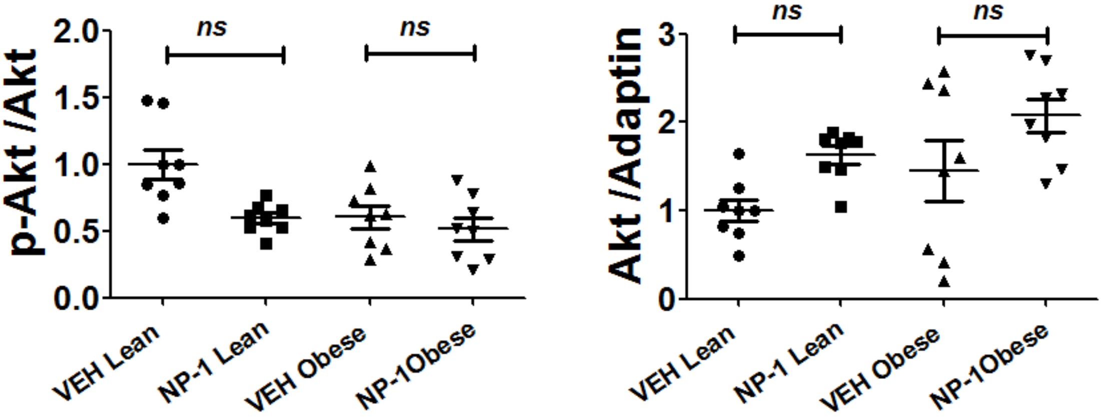

Supplementary Figure 2. Effect of chronic NP-1 treatment on the expression of phosphorylated form of Akt (p-Akt) and total Akt in skeletal muscle of lean and obese rats. Histograms depict the ratios for p-Akt/Akt and Akt /adaptin determined through densitometry. Four samples per group were derived at the same time and processed in parallel in the same inmmunoblot. Histograms represent data from 2 independent inmmunoblots (n = 8 samples per group). Adaptin was used as loading control per lane for muscle samples. The adjustment to digital images did not alter the information contained therein. The data represent the means ± SEM of samples from 8 animals per treatment and genotype. The significance of differences between groups was evaluated using two-way ANOVA and Bonferroni post-hoc test. *ns*= no significant

Supplementary Table S1. Plasma metabolites in lean and obese Zucker rats after a 15-day exposure to VEH or NP-1 (5 mg Kg^{-1}, daily, i.p.).

	**Lean**		**Obese**		**Two way ANOVA**		
**Metabolite**	**VEH**	**NP-1**	**VEH**	**NP-1**	**Interaction**	**Genotype**	**Treatment**
**Urea (mg/dL)**	34.50±1.16	33.71±1.36	33.50±0.53	29.88±1.82	NS	NS	NS
**Uric Acid (mg/dL)**	0.68±0.04	0.67±0.02	0.71±0.03	0.72±0.02	NS	NS	NS
**Creatinine (mg/dL)**	0.72±0.04	0.72±0.04	0.89±0.01**	0.95±0.02$^{###}$	NS	*P*<0.0001	NS
**GGT (IU)**	9.67±1.12	7.00±0.72	15.00±1.14**	19.13±1.10$^{###\$}$	*P*=0.0036	*P*<0.0001	NS
**AST (IU)**	113.83±6.0[illegible]	117.29±3.41	175.63±10.93***	163.88±9.80$^{##}$	NS	*P*<0.0001	NS
**ALT (IU)**	43.83±1.25	37.71±4.38	72.38±5.18***	68.00±1.11$^{###}$	NS	*P*<0.0001	NS
**AST/ALT (IU)**	2.44±0.14	3.35±0.36	2.51±0.25	2.41±0.13$^{#}$	*P*=0.0474	NS	NS

Values are expressed as means ± SEM. Statistical analysis was performed by two-way ANOVA with genotype and treatment as factors (n=6-9 rats per group). Interaction between both factors was also studied. NS: non-significant. Tukey's multiple comparisons indicated significant differences between groups: ***P* <0.01 and ****P* <0.001 *vs* VEH lean. $^{#}$*P* <0.05, $^{##}$*P* <0.01 and $^{###}$*P* <0.001 *vs* NP-1 lean. $^{\$}$*P* <0.05 *vs* obese NP-1.

Supplementary Table S2. Primer references for TaqMan® Gene Expression Assays (Applied Biosystems).

Gene description	Assay ID	Nº accession GenBank	Amplicon Length
**Target genes**			
Adipoq	Rn00595250_m1	NM_144744.3	63
AdipoR1	Rn01483784_m1	NM_207587.1	94
AdipoR2	Rn01463173_m1	NM_001037979.1	83
Dnmt1	Rn00709664_m1	NM_053354.3	62
Ucp1	Rn00562126_m1	NM_001033694.1	69
Hmgcr	Rn00565598_m1	NM_013134.2	71
Cpt1a	Rn00580702_m1	NM_031559.2	64
**Reference genes**			
Actb	Rn00667869_m1	NM_031144.2	91
Gapdh	Rn01775763_g1	NC_005103.4	174
Rpl19	Rn00821265_g1	NM_031103.1	57
Sp1	Rn00561953_m1	NM_012655.2	87
